# 3,4-Dibromo-2,5-bis­[(dieth­oxy­phosphor­yl)meth­yl]-1-phenyl­sulfonyl-1*H*-pyrrole

**DOI:** 10.1107/S1600536811030273

**Published:** 2011-08-02

**Authors:** S. Karthikeyan, K. Sethusankar, Ganesan Gobi Rajeswaran, Arasambattu K. Mohanakrishnan

**Affiliations:** a Department of Physics, RKM Vivekananda College (Autonomous), Chennai 600 004, India; b Department of Organic Chemistry, University of Madras, Maraimalai Campus, Chennai 600 025, India

## Abstract

In the title compound, C_20_H_29_Br_2_NO_8_P_2_S, the pyrrole ring is essentially planar, with a maximum deviation of 0.013 (3) Å for a C atom. The pyrrole ring is almost orthogonal to the sulfonyl-bound phenyl ring, with a dihedral angle 88.5 (2)°. Both P atoms exhibit distorted tetra­hedral configurations with O—P—O angles widened and O—P—C angles narrowed from the ideal tetra­hedral value. In the crystal, mol­ecules are linked into centrosymmetric dimers *via* C—H⋯O inter­actions, resulting in *R*_2_^2^(10) graph-set motifs which are further consolidated by *R*_2_^2^(13) graph-set ring motifs *via* C—H⋯O inter­actions, further resulting in chains of mol­ecules running parallel to the *c* axis; a phosphono O atom is involved in bifurcated hydrogen bonding. All the eth­oxy groups are disordered over two positions each with unequal site-occupancy factors.

## Related literature

For a related structure, see: Seshadri *et al.* (2009 ▶). For applications of pyrrole derivatives, see: Faulkner (2002 ▶); Banwell *et al.* (2006 ▶); Fabio *et al.* (2007 ▶). For comparison of mol­ecular dimensions, see: Bassindale (1984 ▶). For graph-set motifs, see: Bernstein *et al.* (1995 ▶).
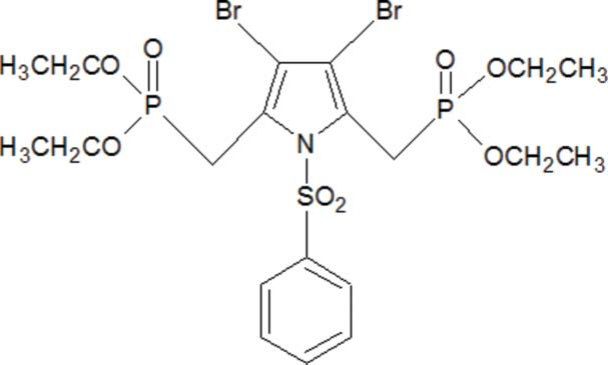


## Experimental

### 

#### Crystal data

C_20_H_29_Br_2_NO_8_P_2_S
*M* *_r_* = 665.26
Monoclinic, 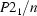

*a* = 9.6524 (2) Å
*b* = 17.5137 (5) Å
*c* = 15.8965 (4) Å
β = 95.506 (1)°
*V* = 2674.89 (12) Å^3^
*Z* = 4
Mo *K*α radiation
μ = 3.27 mm^−1^
*T* = 293 K
0.30 × 0.25 × 0.20 mm

#### Data collection

Bruker Kappa APEXII CCD diffractometer
Absorption correction: multi-scan (*SADABS*; Sheldrick, 1996 ▶) *T*_min_ = 0.440, *T*_max_ = 0.561
34672 measured reflections
8138 independent reflections
4819 reflections with *I* > 2σ(*I*)
*R*_int_ = 0.039

#### Refinement

*R*[*F* ^2^ > 2σ(*F* ^2^)] = 0.045
*wR*(*F* ^2^) = 0.121
*S* = 1.00
8138 reflections
355 parameters
20 restraints
H-atom parameters constrained
Δρ_max_ = 0.79 e Å^−3^
Δρ_min_ = −0.76 e Å^−3^

### 

Data collection: *APEX2* (Bruker, 2004 ▶); cell refinement: *SAINT* (Bruker, 2004 ▶); data reduction: *SAINT*; program(s) used to solve structure: *SHELXS97* (Sheldrick, 2008 ▶); program(s) used to refine structure: *SHELXL97* (Sheldrick, 2008 ▶); molecular graphics: *ORTEP-3* (Farrugia, 1997 ▶); software used to prepare material for publication: *SHELXL97* and *PLATON* (Spek, 2009 ▶).

## Supplementary Material

Crystal structure: contains datablock(s) global, I. DOI: 10.1107/S1600536811030273/pv2427sup1.cif

Structure factors: contains datablock(s) I. DOI: 10.1107/S1600536811030273/pv2427Isup2.hkl

Supplementary material file. DOI: 10.1107/S1600536811030273/pv2427Isup3.cml

Additional supplementary materials:  crystallographic information; 3D view; checkCIF report

## Figures and Tables

**Table 1:** Hydrogen-bond geometry (Å, °)

*D*—H⋯*A*	*D*—H	H⋯*A*	*D*⋯*A*	*D*—H⋯*A*
C5—H5*A*⋯O6^i^	0.97	2.34	3.272 (4)	162
C16—H16⋯O3^ii^	0.93	2.44	3.162 (5)	134
C11—H11*B*⋯O6^iii^	0.97	2.62	3.592 (19)	179

Symmetry codes: (i) 
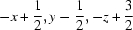
; (ii) 
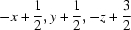
; (iii) 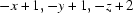.

**Fig. 1.**
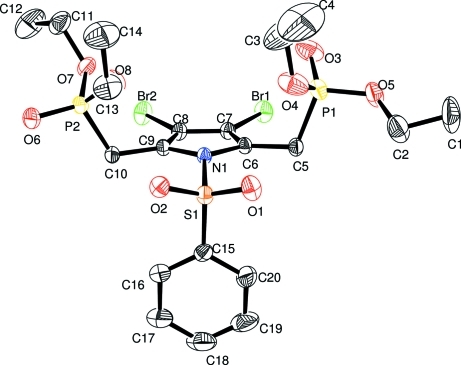
The molecular structure of the title compound showing only the atoms representing major fractions of the disordered ethoxy groups, with the atom numbering scheme. Displacement ellipsoids are drawn at 30% probability level.

**Fig. 2.**
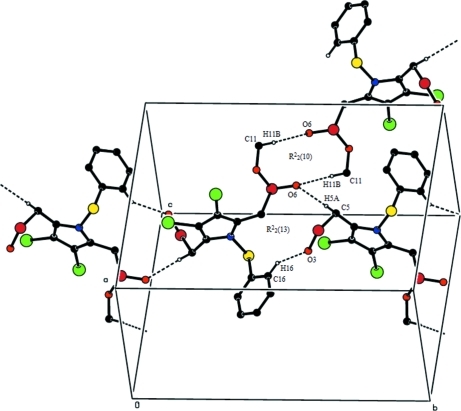
The crystal packing of the title compound viewed down a axis, showing the hydrogen bonds resulting in R22(10) and R22(13) graph-set ring motifs; H-atoms not involved in H-bonds have been excluded for clarity.

## supplementary crystallographic information

### Comment

Pyrrole derivatives are known to occur in many marine organisms (Faulkner, 2002). Pyrrole and its derivatives have shown to possess biological activities such as antibacterial, antiallergic, antitumor (Banwell, *et al.*, 2006), and some of them are well known as metabotropic receptor antagonists (Fabio, *et al.*., 2007).

In the title compound (Fig. 1), the pyrrole ring is essentially planar with a maximum deviation of 0.013 (3)Å for atom C9. The methyl carbon atoms C5 and C10 and S1 atom lie -0.078 (3), 0.034 (3) and 0.5396 (8) Å, respectively, from the pyrrole plane. The pyrrole ring is almost orthogonal to the sulfonyl bound phenyl ring, with a dihedral angle 88.5 (2)°.

Atoms P1 and P2 have distorted tetrahedral configurations. The widening of angles O3—P1—O4 [114.4 (13)°] and O6—P2—O7 [120.0 (7)°] and narrowing of angles O5—P1—C5 [95.0 (9)°] and O8—P2—C10 [107.4 (9)°] from the ideal tetrahedral value are attributed to the Thrope-Ingold effect (Bassindale, 1984). The ethoxy side chains were disordered with un-equal site occupancy factors.

The crystal packing is stabilized by C—H···O intermolecular interactions. The molecules are linked into centrosymmetric dimers *via* C11—H11B···O6 interactions resulting in *R*^2^_2_(10) graph set motif (Bernstein, *et al.*, 1995). The dimers are further consolidated by *R*^2^_2_(13) graph-set ring motif *via* C5—H5A···O6 and C16—H16···O3 interactions resulting in chains of molecules running parallel to the *c*-axis (Tab. 1 & Fig. 2); the phosphono O atom (O6) is involved in bifurcated hydrogen bonding.

### Experimental

To a solution of 3,4-dibromo-2,5-bis(bromomethyl)-1-phenylsulfonyl- pyrrole (1 mmol) and triethylphosphite (2.5 mmol) in dry dichloromethane at room temperature, ZnBr_2_ (0.4 mmol) was added and allowed to stir for 4 h under N_2_. After consumption of the 3,4-dibromo-2,5-bis (bromomethyl)-1-phenylsulfonyl-pyrrole (monitored by TLC) volatile components were removed under vacuo. The residual mass was poured over crushed ice containing conc. HCl. The precipitated solid was filtered, washed with water and dried to give crude phosphonate ester which was crystallized from CHCl_3_.

### Refinement

The ethoxy groups were disordered over positions C4/C3/O4:C4'/C3'/O4', C1/C2/O5:C1'/C2'/O5', C12/C11/O7:C12'/C11'/O7' and C14/C13/O8:C14'/C13'/O8' with site occupancy factors 0.411 (10):0.589 (10), 0.121 (5):0.879 (5), 0.609 (13):0.391 (13) and 0.719 (8):0.281 (8), respectively. The distance P–O and P–O' were restrained to be equal with an esd value 0.01. The C–C and O–C bond lengths were restrained at C–C = 1.54 (1) and O–C = 1.45 (1) Å. The anisotropic thermal parameter of the atom pairs were treated equally in the refinement. All the hydrogen atoms of the compound were fixed geometrically and allowed to ride on their parent atoms with C–H distance in the range 0.93 to 0.97Å and with *U*_iso_(H) = 1.5*U*_eq_(C) for CH_3_ groups and *U*_iso_(H) = 1.2*U*_eq_(C) for all the other groups.

### Crystal data

**Table:**

C_20_H_29_Br_2_NO_8_P_2_S	*F*(000) = 1344
*M**_r_* = 665.26	*D*_x_ = 1.652 Mg m^−^^3^
Monoclinic, *P*2_1_/*n*	Mo *K*α radiation, λ = 0.71073 Å
Hall symbol: -P 2yn	Cell parameters from 8138 reflections
*a* = 9.6524 (2) Å	θ = 2.3–30.5°
*b* = 17.5137 (5) Å	µ = 3.27 mm^−^^1^
*c* = 15.8965 (4) Å	*T* = 293 K
β = 95.506 (1)°	Block, colourless
*V* = 2674.89 (12) Å^3^	0.30 × 0.25 × 0.20 mm
*Z* = 4	

### Data collection

**Table:**

Bruker Kappa APEXII CCD diffractometer	8138 independent reflections
Radiation source: fine-focus sealed tube	4819 reflections with *I* > 2σ(*I*)
graphite	*R*_int_ = 0.039
ω scans	θ_max_ = 30.5°, θ_min_ = 2.3°
Absorption correction: multi-scan (*SADABS*; Sheldrick, 1996)	*h* = −13→13
*T*_min_ = 0.440, *T*_max_ = 0.561	*k* = −24→24
34672 measured reflections	*l* = −22→22

### Refinement

**Table:**

Refinement on *F*^2^	Primary atom site location: structure-invariant direct methods
Least-squares matrix: full	Secondary atom site location: difference Fourier map
*R*[*F*^2^ > 2σ(*F*^2^)] = 0.045	Hydrogen site location: inferred from neighbouring sites
*wR*(*F*^2^) = 0.121	H-atom parameters constrained
*S* = 1.00	*w* = 1/[σ^2^(*F*_o_^2^) + (0.0558*P*)^2^ + 1.4803*P*] where *P* = (*F*_o_^2^ + 2*F*_c_^2^)/3
8138 reflections	(Δ/σ)_max_ = 0.002
355 parameters	Δρ_max_ = 0.79 e Å^−^^3^
20 restraints	Δρ_min_ = −0.76 e Å^−^^3^

### Special details

**Table:**

Geometry. All e.s.d.'s (except the e.s.d. in the dihedral angle between two l.s. planes) are estimated using the full covariance matrix. The cell e.s.d.'s are taken into account individually in the estimation of e.s.d.'s in distances, angles and torsion angles; correlations between e.s.d.'s in cell parameters are only used when they are defined by crystal symmetry. An approximate (isotropic) treatment of cell e.s.d.'s is used for estimating e.s.d.'s involving l.s. planes.
Refinement. Refinement of *F*^2^ against ALL reflections. The weighted *R*-factor *wR* and goodness of fit *S* are based on *F*^2^, conventional *R*-factors *R* are based on *F*, with *F* set to zero for negative *F*^2^. The threshold expression of *F*^2^ > σ(*F*^2^) is used only for calculating *R*-factors(gt) *etc*. and is not relevant to the choice of reflections for refinement. *R*-factors based on *F*^2^ are statistically about twice as large as those based on *F*, and *R*- factors based on ALL data will be even larger.

### Fractional atomic coordinates and isotropic or equivalent isotropic displacement parameters (Å^2^)

**Table:**

	*x*	*y*	*z*	*U*_iso_*/*U*_eq_	Occ. (<1)
C5	0.0820 (3)	0.13111 (17)	0.74030 (19)	0.0400 (7)	
H5A	0.0917	0.0934	0.6965	0.048*	
H5B	−0.0043	0.1585	0.7257	0.048*	
C6	0.2004 (3)	0.18591 (16)	0.74214 (17)	0.0335 (6)	
C7	0.3347 (3)	0.16883 (16)	0.73313 (17)	0.0357 (6)	
C8	0.4160 (3)	0.23518 (17)	0.74534 (18)	0.0359 (6)	
C9	0.3326 (3)	0.29465 (16)	0.76079 (17)	0.0337 (6)	
C10	0.3748 (3)	0.37604 (17)	0.77607 (18)	0.0372 (6)	
H10A	0.2975	0.4082	0.7546	0.045*	
H10B	0.4514	0.3869	0.7426	0.045*	
C15	0.0367 (3)	0.33865 (18)	0.6355 (2)	0.0452 (7)	
C16	0.1028 (4)	0.4005 (2)	0.6062 (2)	0.0591 (9)	
H16	0.1580	0.4316	0.6432	0.071*	
C17	0.0868 (5)	0.4165 (3)	0.5209 (3)	0.0812 (13)	
H17	0.1331	0.4580	0.5000	0.097*	
C18	0.0039 (7)	0.3722 (3)	0.4671 (3)	0.0974 (18)	
H18	−0.0063	0.3835	0.4097	0.117*	
C19	−0.0652 (7)	0.3108 (3)	0.4970 (3)	0.106 (2)	
H19	−0.1228	0.2811	0.4599	0.127*	
C20	−0.0490 (5)	0.2931 (2)	0.5821 (3)	0.0767 (13)	
H20	−0.0948	0.2515	0.6029	0.092*	
N1	0.1966 (2)	0.26458 (13)	0.76122 (15)	0.0329 (5)	
O1	−0.0609 (2)	0.27254 (14)	0.76316 (18)	0.0588 (6)	
O2	0.0806 (2)	0.38764 (13)	0.78932 (14)	0.0492 (5)	
O3	0.2002 (3)	0.04497 (19)	0.8743 (2)	0.0855 (9)	
O6	0.4468 (3)	0.48635 (13)	0.89029 (15)	0.0642 (7)	
P1	0.07187 (10)	0.08262 (5)	0.83967 (6)	0.0522 (2)	
P2	0.42646 (9)	0.40435 (5)	0.88263 (5)	0.0430 (2)	
S1	0.05156 (8)	0.31902 (4)	0.74386 (5)	0.03984 (18)	
Br1	0.40069 (4)	0.071636 (19)	0.71196 (2)	0.05379 (12)	
Br2	0.60841 (3)	0.23995 (2)	0.74283 (3)	0.05732 (12)	
O5	−0.0926 (10)	0.0770 (13)	0.8306 (16)	0.0668 (9)	0.121 (5)
C1	−0.248 (5)	−0.029 (3)	0.741 (3)	0.124 (3)	0.121 (5)
H1A	−0.3481	−0.0284	0.7344	0.186*	0.121 (5)
H1B	−0.2161	−0.0809	0.7469	0.186*	0.121 (5)
H1C	−0.2134	−0.0066	0.6921	0.186*	0.121 (5)
C2	−0.195 (5)	0.017 (3)	0.820 (4)	0.098 (2)	0.121 (5)
H2A	−0.2785	0.0386	0.8403	0.117*	0.121 (5)
H2B	−0.1631	−0.0216	0.8616	0.117*	0.121 (5)
O5'	−0.0402 (3)	0.01914 (17)	0.8237 (2)	0.0668 (9)	0.879 (5)
C1'	−0.2651 (6)	−0.0326 (4)	0.8086 (7)	0.124 (3)	0.879 (5)
H1'1	−0.2206	−0.0769	0.7880	0.186*	0.879 (5)
H1'2	−0.3563	−0.0272	0.7794	0.186*	0.879 (5)
H1'3	−0.2727	−0.0381	0.8681	0.186*	0.879 (5)
C2'	−0.1804 (6)	0.0369 (4)	0.7937 (5)	0.098 (2)	0.879 (5)
H2'1	−0.1871	0.0491	0.7339	0.117*	0.879 (5)
H2'2	−0.2131	0.0803	0.8240	0.117*	0.879 (5)
O4	0.009 (5)	0.1382 (13)	0.9016 (12)	0.078 (4)	0.411 (10)
C4	−0.007 (3)	0.1903 (13)	1.0324 (17)	0.173 (7)	0.411 (10)
H4A	0.0866	0.2090	1.0441	0.260*	0.411 (10)
H4B	−0.0482	0.1842	1.0845	0.260*	0.411 (10)
H4C	−0.0603	0.2260	0.9970	0.260*	0.411 (10)
C3	−0.004 (2)	0.1141 (11)	0.9879 (11)	0.125 (4)	0.411 (10)
H3A	0.0749	0.0835	1.0101	0.150*	0.411 (10)
H3B	−0.0892	0.0857	0.9923	0.150*	0.411 (10)
O4'	0.028 (3)	0.1505 (8)	0.8938 (8)	0.078 (4)	0.589 (10)
C3'	0.0613 (13)	0.1538 (9)	0.9850 (7)	0.125 (4)	0.589 (10)
H3'1	0.1069	0.2019	0.9999	0.150*	0.589 (10)
H3'2	0.1257	0.1130	1.0025	0.150*	0.589 (10)
C4'	−0.0661 (17)	0.1464 (14)	1.0318 (11)	0.173 (7)	0.589 (10)
H4'1	−0.1202	0.1035	1.0101	0.260*	0.589 (10)
H4'2	−0.1208	0.1921	1.0242	0.260*	0.589 (10)
H4'3	−0.0386	0.1389	1.0908	0.260*	0.589 (10)
O8	0.318 (2)	0.3710 (6)	0.9390 (13)	0.0637 (10)	0.719 (8)
C13	0.2084 (9)	0.4151 (5)	0.9727 (4)	0.078 (2)	0.719 (8)
H13A	0.1178	0.3962	0.9503	0.094*	0.719 (8)
H13B	0.2155	0.4684	0.9569	0.094*	0.719 (8)
C14	0.2251 (11)	0.4074 (6)	1.0654 (4)	0.115 (3)	0.719 (8)
H14A	0.2137	0.3548	1.0804	0.173*	0.719 (8)
H14B	0.1561	0.4378	1.0894	0.173*	0.719 (8)
H14C	0.3162	0.4244	1.0868	0.173*	0.719 (8)
O8'	0.322 (6)	0.3676 (15)	0.940 (3)	0.0637 (10)	0.281 (8)
C13'	0.278 (2)	0.4208 (13)	1.0017 (14)	0.078 (2)	0.281 (8)
H13C	0.2589	0.4709	0.9772	0.094*	0.281 (8)
H13D	0.3467	0.4254	1.0499	0.094*	0.281 (8)
C14'	0.145 (2)	0.3836 (16)	1.0264 (14)	0.115 (3)	0.281 (8)
H14D	0.0802	0.4224	1.0392	0.173*	0.281 (8)
H14E	0.1668	0.3519	1.0752	0.173*	0.281 (8)
H14F	0.1048	0.3529	0.9803	0.173*	0.281 (8)
O7	0.5503 (12)	0.3488 (9)	0.9077 (17)	0.054 (3)	0.609 (13)
C12	0.7959 (11)	0.3707 (10)	0.9575 (9)	0.120 (4)	0.609 (13)
H12A	0.8302	0.3249	0.9335	0.180*	0.609 (13)
H12B	0.8557	0.3845	1.0069	0.180*	0.609 (13)
H12C	0.7943	0.4112	0.9168	0.180*	0.609 (13)
C11	0.6496 (16)	0.3569 (8)	0.9817 (15)	0.079 (3)	0.609 (13)
H11A	0.6488	0.3110	1.0158	0.095*	0.609 (13)
H11B	0.6221	0.3994	1.0156	0.095*	0.609 (13)
O7'	0.569 (2)	0.3624 (16)	0.900 (3)	0.054 (3)	0.391 (13)
C11'	0.648 (3)	0.3788 (13)	0.980 (2)	0.079 (3)	0.391 (13)
H11C	0.5906	0.3750	1.0269	0.095*	0.391 (13)
H11D	0.6889	0.4295	0.9803	0.095*	0.391 (13)
C12'	0.7607 (19)	0.3174 (15)	0.9864 (14)	0.120 (4)	0.391 (13)
H12D	0.7218	0.2700	1.0031	0.180*	0.391 (13)
H12E	0.8352	0.3323	1.0275	0.180*	0.391 (13)
H12F	0.7959	0.3114	0.9323	0.180*	0.391 (13)

### Atomic displacement parameters (Å^2^)

**Table:**

	*U*^11^	*U*^22^	*U*^33^	*U*^12^	*U*^13^	*U*^23^
C5	0.0387 (16)	0.0307 (15)	0.0499 (17)	−0.0039 (12)	−0.0003 (13)	−0.0013 (13)
C6	0.0332 (14)	0.0281 (14)	0.0393 (15)	0.0000 (11)	0.0035 (11)	−0.0014 (11)
C7	0.0373 (15)	0.0300 (14)	0.0400 (15)	0.0027 (12)	0.0052 (12)	−0.0054 (12)
C8	0.0257 (13)	0.0400 (16)	0.0430 (15)	−0.0003 (12)	0.0082 (11)	−0.0025 (13)
C9	0.0332 (14)	0.0296 (14)	0.0384 (15)	−0.0031 (12)	0.0031 (11)	−0.0008 (11)
C10	0.0357 (15)	0.0328 (15)	0.0423 (16)	−0.0052 (12)	−0.0002 (12)	0.0007 (12)
C15	0.0425 (17)	0.0370 (17)	0.0534 (18)	0.0123 (14)	−0.0096 (14)	−0.0056 (14)
C16	0.059 (2)	0.061 (2)	0.055 (2)	−0.0022 (19)	−0.0081 (17)	0.0075 (18)
C17	0.090 (3)	0.087 (3)	0.063 (3)	0.004 (3)	−0.006 (2)	0.017 (2)
C18	0.151 (5)	0.077 (3)	0.057 (3)	0.033 (4)	−0.025 (3)	−0.001 (2)
C19	0.158 (6)	0.071 (3)	0.075 (3)	0.020 (3)	−0.058 (3)	−0.020 (3)
C20	0.094 (3)	0.042 (2)	0.086 (3)	−0.001 (2)	−0.029 (2)	−0.006 (2)
N1	0.0310 (11)	0.0248 (11)	0.0426 (13)	0.0023 (9)	0.0013 (10)	−0.0015 (10)
O1	0.0335 (11)	0.0501 (14)	0.0946 (19)	0.0041 (10)	0.0150 (12)	0.0046 (13)
O2	0.0519 (13)	0.0376 (12)	0.0579 (14)	0.0112 (10)	0.0040 (11)	−0.0096 (10)
O3	0.079 (2)	0.086 (2)	0.091 (2)	0.0235 (17)	0.0097 (17)	0.0353 (18)
O6	0.098 (2)	0.0348 (13)	0.0568 (14)	−0.0080 (13)	−0.0081 (14)	−0.0076 (11)
P1	0.0545 (5)	0.0449 (5)	0.0587 (5)	−0.0079 (4)	0.0124 (4)	0.0034 (4)
P2	0.0529 (5)	0.0307 (4)	0.0442 (4)	−0.0004 (4)	−0.0008 (4)	−0.0029 (3)
S1	0.0312 (4)	0.0324 (4)	0.0559 (4)	0.0053 (3)	0.0039 (3)	−0.0020 (3)
Br1	0.0543 (2)	0.03644 (18)	0.0716 (2)	0.01011 (15)	0.01144 (17)	−0.01260 (15)
Br2	0.03251 (17)	0.0609 (2)	0.0805 (3)	−0.00089 (15)	0.01528 (16)	−0.01378 (18)
O5	0.0502 (17)	0.0469 (18)	0.105 (2)	−0.0058 (13)	0.0133 (16)	0.0164 (16)
C1	0.058 (3)	0.078 (4)	0.233 (10)	−0.026 (3)	−0.005 (5)	0.030 (6)
C2	0.052 (3)	0.081 (4)	0.158 (7)	0.003 (3)	0.001 (4)	0.042 (4)
O5'	0.0502 (17)	0.0469 (18)	0.105 (2)	−0.0058 (13)	0.0133 (16)	0.0164 (16)
C1'	0.058 (3)	0.078 (4)	0.233 (10)	−0.026 (3)	−0.005 (5)	0.030 (6)
C2'	0.052 (3)	0.081 (4)	0.158 (7)	0.003 (3)	0.001 (4)	0.042 (4)
O4	0.118 (8)	0.067 (5)	0.053 (3)	0.015 (6)	0.031 (3)	0.006 (3)
C4	0.176 (17)	0.26 (2)	0.088 (5)	−0.056 (11)	0.057 (9)	−0.045 (12)
C3	0.134 (11)	0.149 (13)	0.093 (5)	−0.023 (7)	0.014 (7)	−0.046 (7)
O4'	0.118 (8)	0.067 (5)	0.053 (3)	0.015 (6)	0.031 (3)	0.006 (3)
C3'	0.134 (11)	0.149 (13)	0.093 (5)	−0.023 (7)	0.014 (7)	−0.046 (7)
C4'	0.176 (17)	0.26 (2)	0.088 (5)	−0.056 (11)	0.057 (9)	−0.045 (12)
O8	0.083 (2)	0.0512 (18)	0.0605 (15)	0.0023 (17)	0.0247 (16)	0.0014 (15)
C13	0.075 (5)	0.096 (4)	0.066 (4)	0.012 (5)	0.020 (3)	−0.005 (4)
C14	0.125 (8)	0.159 (8)	0.065 (5)	0.005 (6)	0.026 (4)	0.001 (5)
O8'	0.083 (2)	0.0512 (18)	0.0605 (15)	0.0023 (17)	0.0247 (16)	0.0014 (15)
C13'	0.075 (5)	0.096 (4)	0.066 (4)	0.012 (5)	0.020 (3)	−0.005 (4)
C14'	0.125 (8)	0.159 (8)	0.065 (5)	0.005 (6)	0.026 (4)	0.001 (5)
O7	0.053 (3)	0.047 (6)	0.057 (4)	0.001 (4)	−0.015 (4)	−0.008 (4)
C12	0.067 (6)	0.157 (13)	0.130 (9)	0.014 (7)	−0.024 (5)	−0.033 (9)
C11	0.089 (4)	0.058 (8)	0.084 (3)	0.011 (5)	−0.028 (3)	−0.021 (7)
O7'	0.053 (3)	0.047 (6)	0.057 (4)	0.001 (4)	−0.015 (4)	−0.008 (4)
C11'	0.089 (4)	0.058 (8)	0.084 (3)	0.011 (5)	−0.028 (3)	−0.021 (7)
C12'	0.067 (6)	0.157 (13)	0.130 (9)	0.014 (7)	−0.024 (5)	−0.033 (9)

### Geometric parameters (Å, °)

**Table:**

C5—C6	1.491 (4)	C1'—C2'	1.497 (6)
C5—P1	1.804 (3)	C1'—H1'1	0.9600
C5—H5A	0.9700	C1'—H1'2	0.9600
C5—H5B	0.9700	C1'—H1'3	0.9600
C6—C7	1.351 (4)	C2'—H2'1	0.9700
C6—N1	1.412 (3)	C2'—H2'2	0.9700
C7—C8	1.405 (4)	O4—C3	1.453 (10)
C7—Br1	1.860 (3)	C4—C3	1.511 (10)
C8—C9	1.353 (4)	C4—H4A	0.9600
C8—Br2	1.863 (3)	C4—H4B	0.9600
C9—N1	1.415 (4)	C4—H4C	0.9600
C9—C10	1.496 (4)	C3—H3A	0.9700
C10—P2	1.789 (3)	C3—H3B	0.9700
C10—H10A	0.9700	O4'—C3'	1.456 (10)
C10—H10B	0.9700	C3'—C4'	1.502 (9)
C15—C16	1.363 (5)	C3'—H3'1	0.9700
C15—C20	1.381 (5)	C3'—H3'2	0.9700
C15—S1	1.748 (3)	C4'—H4'1	0.9600
C16—C17	1.379 (6)	C4'—H4'2	0.9600
C16—H16	0.9300	C4'—H4'3	0.9600
C17—C18	1.357 (7)	O8—C13	1.451 (10)
C17—H17	0.9300	C13—C14	1.473 (7)
C18—C19	1.374 (8)	C13—H13A	0.9700
C18—H18	0.9300	C13—H13B	0.9700
C19—C20	1.382 (7)	C14—H14A	0.9600
C19—H19	0.9300	C14—H14B	0.9600
C20—H20	0.9300	C14—H14C	0.9600
N1—S1	1.694 (2)	O8'—C13'	1.450 (10)
O1—S1	1.414 (3)	C13'—C14'	1.519 (10)
O2—S1	1.416 (2)	C13'—H13C	0.9700
O3—P1	1.463 (3)	C13'—H13D	0.9700
O6—P2	1.453 (2)	C14'—H14D	0.9600
P1—O4	1.548 (7)	C14'—H14E	0.9600
P1—O4'	1.549 (6)	C14'—H14F	0.9600
P1—O5'	1.555 (3)	O7—C11	1.452 (7)
P1—O5	1.583 (10)	C12—C11	1.518 (10)
P2—O8'	1.558 (8)	C12—H12A	0.9600
P2—O8	1.558 (4)	C12—H12B	0.9600
P2—O7'	1.561 (7)	C12—H12C	0.9600
P2—O7	1.563 (5)	C11—H11A	0.9700
O5—C2	1.447 (10)	C11—H11B	0.9700
O5—O4	1.78 (4)	O7'—C11'	1.448 (9)
C1—C2	1.539 (10)	C11'—C12'	1.524 (10)
C1—H1A	0.9600	C11'—H11C	0.9700
C1—H1B	0.9600	C11'—H11D	0.9700
C1—H1C	0.9600	C12'—H12D	0.9600
C2—H2A	0.9700	C12'—H12E	0.9600
C2—H2B	0.9700	C12'—H12F	0.9600
O5'—C2'	1.425 (6)		
			
C6—C5—P1	113.1 (2)	O5—C2—H2A	104.8
C6—C5—H5A	109.0	C1—C2—H2A	104.8
P1—C5—H5A	109.0	O5—C2—H2B	104.8
C6—C5—H5B	109.0	C1—C2—H2B	104.8
P1—C5—H5B	109.0	H2A—C2—H2B	105.8
H5A—C5—H5B	107.8	C2'—O5'—P1	121.5 (3)
C7—C6—N1	106.5 (2)	C2'—C1'—H1'1	109.5
C7—C6—C5	126.7 (3)	C2'—C1'—H1'2	109.5
N1—C6—C5	126.6 (2)	H1'1—C1'—H1'2	109.5
C6—C7—C8	109.3 (2)	C2'—C1'—H1'3	109.5
C6—C7—Br1	124.8 (2)	H1'1—C1'—H1'3	109.5
C8—C7—Br1	125.8 (2)	H1'2—C1'—H1'3	109.5
C9—C8—C7	109.1 (2)	O5'—C2'—C1'	106.6 (5)
C9—C8—Br2	125.5 (2)	O5'—C2'—H2'1	110.4
C7—C8—Br2	125.4 (2)	C1'—C2'—H2'1	110.4
C8—C9—N1	106.4 (2)	O5'—C2'—H2'2	110.4
C8—C9—C10	127.3 (3)	C1'—C2'—H2'2	110.4
N1—C9—C10	126.3 (3)	H2'1—C2'—H2'2	108.6
C9—C10—P2	117.7 (2)	C3—O4—P1	119.6 (13)
C9—C10—H10A	107.9	C3—O4—O5	109 (2)
P2—C10—H10A	107.9	P1—O4—O5	56.3 (10)
C9—C10—H10B	107.9	O4—C3—C4	101.2 (16)
P2—C10—H10B	107.9	O4—C3—H3A	111.5
H10A—C10—H10B	107.2	C4—C3—H3A	111.5
C16—C15—C20	121.4 (4)	O4—C3—H3B	111.5
C16—C15—S1	119.8 (3)	C4—C3—H3B	111.5
C20—C15—S1	118.7 (3)	H3A—C3—H3B	109.3
C15—C16—C17	119.1 (4)	C3'—O4'—P1	122.6 (11)
C15—C16—H16	120.5	O4'—C3'—C4'	112.3 (14)
C17—C16—H16	120.5	O4'—C3'—H3'1	109.1
C18—C17—C16	120.4 (5)	C4'—C3'—H3'1	109.1
C18—C17—H17	119.8	O4'—C3'—H3'2	109.1
C16—C17—H17	119.8	C4'—C3'—H3'2	109.1
C17—C18—C19	120.4 (4)	H3'1—C3'—H3'2	107.9
C17—C18—H18	119.8	C3'—C4'—H4'1	109.5
C19—C18—H18	119.8	C3'—C4'—H4'2	109.5
C18—C19—C20	120.0 (4)	H4'1—C4'—H4'2	109.5
C18—C19—H19	120.0	C3'—C4'—H4'3	109.5
C20—C19—H19	120.0	H4'1—C4'—H4'3	109.5
C15—C20—C19	118.5 (5)	H4'2—C4'—H4'3	109.5
C15—C20—H20	120.7	C13—O8—P2	124.5 (9)
C19—C20—H20	120.7	O8—C13—C14	107.8 (11)
C6—N1—C9	108.6 (2)	O8—C13—H13A	110.1
C6—N1—S1	123.55 (19)	C14—C13—H13A	110.1
C9—N1—S1	122.83 (19)	O8—C13—H13B	110.1
O3—P1—O4	114.4 (13)	C14—C13—H13B	110.1
O3—P1—O4'	113.7 (10)	H13A—C13—H13B	108.5
O3—P1—O5'	106.83 (19)	C13'—O8'—P2	112.4 (14)
O4—P1—O5'	104.2 (17)	O8'—C13'—C14'	102.2 (17)
O4'—P1—O5'	114.7 (11)	O8'—C13'—H13C	111.3
O3—P1—O5	144.2 (8)	C14'—C13'—H13C	111.3
O4—P1—O5	69 (2)	O8'—C13'—H13D	111.3
O4'—P1—O5	77.1 (15)	C14'—C13'—H13D	111.3
O3—P1—C5	115.23 (17)	H13C—C13'—H13D	109.2
O4—P1—C5	108.5 (8)	C13'—C14'—H14D	109.5
O4'—P1—C5	99.5 (5)	C13'—C14'—H14E	109.5
O5'—P1—C5	106.80 (16)	H14D—C14'—H14E	109.5
O5—P1—C5	95.0 (9)	C13'—C14'—H14F	109.5
O6—P2—O8'	116.7 (9)	H14D—C14'—H14F	109.5
O6—P2—O8	114.5 (4)	H14E—C14'—H14F	109.5
O6—P2—O7'	109.9 (13)	C11—O7—P2	125.1 (15)
O8'—P2—O7'	108 (3)	O7—C11—C12	111.6 (13)
O8—P2—O7'	110.5 (17)	O7—C11—H11A	109.3
O6—P2—O7	120.0 (7)	C12—C11—H11A	109.3
O8'—P2—O7	97 (2)	O7—C11—H11B	109.3
O8—P2—O7	99.3 (12)	C12—C11—H11B	109.3
O6—P2—C10	112.10 (15)	H11A—C11—H11B	108.0
O8'—P2—C10	107 (2)	C11'—O7'—P2	117 (2)
O8—P2—C10	107.4 (9)	O7'—C11'—C12'	103.2 (16)
O7'—P2—C10	101.7 (16)	O7'—C11'—H11C	111.1
O7—P2—C10	101.9 (10)	C12'—C11'—H11C	111.1
O1—S1—O2	119.75 (15)	O7'—C11'—H11D	111.1
O1—S1—N1	106.26 (13)	C12'—C11'—H11D	111.1
O2—S1—N1	105.94 (13)	H11C—C11'—H11D	109.1
O1—S1—C15	109.56 (16)	C11'—C12'—H12D	109.5
O2—S1—C15	109.22 (15)	C11'—C12'—H12E	109.5
N1—S1—C15	105.04 (13)	H12D—C12'—H12E	109.5
C2—O5—P1	137 (3)	C11'—C12'—H12F	109.5
C2—O5—O4	147 (3)	H12D—C12'—H12F	109.5
P1—O5—O4	54.4 (11)	H12E—C12'—H12F	109.5
O5—C2—C1	130 (4)		
			
P1—C5—C6—C7	81.3 (3)	C5—P1—O5—C2	114 (4)
P1—C5—C6—N1	−92.5 (3)	O3—P1—O5—O4	103.4 (16)
N1—C6—C7—C8	−0.5 (3)	O4'—P1—O5—O4	−9.3 (14)
C5—C6—C7—C8	−175.4 (3)	O5'—P1—O5—O4	142.6 (16)
N1—C6—C7—Br1	177.33 (19)	C5—P1—O5—O4	−107.9 (10)
C5—C6—C7—Br1	2.5 (4)	P1—O5—C2—C1	−78 (7)
C6—C7—C8—C9	−1.0 (3)	O4—O5—C2—C1	−172 (4)
Br1—C7—C8—C9	−178.8 (2)	O3—P1—O5'—C2'	176.9 (5)
C6—C7—C8—Br2	177.9 (2)	O4—P1—O5'—C2'	55.5 (11)
Br1—C7—C8—Br2	0.0 (4)	O4'—P1—O5'—C2'	50.0 (8)
C7—C8—C9—N1	2.0 (3)	O5—P1—O5'—C2'	19.6 (14)
Br2—C8—C9—N1	−176.8 (2)	C5—P1—O5'—C2'	−59.3 (5)
C7—C8—C9—C10	−179.0 (3)	P1—O5'—C2'—C1'	−164.6 (5)
Br2—C8—C9—C10	2.2 (4)	O3—P1—O4—C3	−47 (4)
C8—C9—C10—P2	−89.0 (3)	O4'—P1—O4—C3	−136 (13)
N1—C9—C10—P2	89.8 (3)	O5'—P1—O4—C3	69 (3)
C20—C15—C16—C17	−1.9 (6)	O5—P1—O4—C3	95 (3)
S1—C15—C16—C17	−178.1 (3)	C5—P1—O4—C3	−177 (3)
C15—C16—C17—C18	1.4 (7)	O3—P1—O4—O5	−141.4 (10)
C16—C17—C18—C19	0.0 (8)	O4'—P1—O4—O5	130 (11)
C17—C18—C19—C20	−0.9 (9)	O5'—P1—O4—O5	−25.1 (10)
C16—C15—C20—C19	1.0 (6)	C5—P1—O4—O5	88.4 (11)
S1—C15—C20—C19	177.3 (4)	C2—O5—O4—C3	9(6)
C18—C19—C20—C15	0.4 (8)	P1—O5—O4—C3	−113.2 (18)
C7—C6—N1—C9	1.8 (3)	C2—O5—O4—P1	123 (6)
C5—C6—N1—C9	176.6 (3)	P1—O4—C3—C4	156 (3)
C7—C6—N1—S1	157.1 (2)	O5—O4—C3—C4	−142.1 (19)
C5—C6—N1—S1	−28.1 (4)	O3—P1—O4'—C3'	−30 (2)
C8—C9—N1—C6	−2.4 (3)	O4—P1—O4'—C3'	66 (9)
C10—C9—N1—C6	178.6 (3)	O5'—P1—O4'—C3'	93 (2)
C8—C9—N1—S1	−157.9 (2)	O5—P1—O4'—C3'	114 (2)
C10—C9—N1—S1	23.1 (4)	C5—P1—O4'—C3'	−153 (2)
C6—C5—P1—O3	−52.7 (3)	P1—O4'—C3'—C4'	−111 (2)
C6—C5—P1—O4	77.1 (18)	O6—P2—O8—C13	22 (2)
C6—C5—P1—O4'	69.2 (12)	O8'—P2—O8—C13	165 (65)
C6—C5—P1—O5'	−171.2 (2)	O7'—P2—O8—C13	147 (2)
C6—C5—P1—O5	146.9 (9)	O7—P2—O8—C13	151 (2)
C9—C10—P2—O6	−172.3 (2)	C10—P2—O8—C13	−103.3 (18)
C9—C10—P2—O8'	−42.9 (19)	P2—O8—C13—C14	−122.5 (19)
C9—C10—P2—O8	−45.7 (8)	O6—P2—O8'—C13'	−9(6)
C9—C10—P2—O7'	70.4 (13)	O8—P2—O8'—C13'	−47 (58)
C9—C10—P2—O7	58.1 (8)	O7'—P2—O8'—C13'	115 (4)
C6—N1—S1—O1	37.7 (3)	O7—P2—O8'—C13'	119 (4)
C9—N1—S1—O1	−170.4 (2)	C10—P2—O8'—C13'	−136 (4)
C6—N1—S1—O2	166.1 (2)	P2—O8'—C13'—C14'	161 (4)
C9—N1—S1—O2	−42.0 (3)	O6—P2—O7—C11	42 (2)
C6—N1—S1—C15	−78.4 (3)	O8'—P2—O7—C11	−84 (2)
C9—N1—S1—C15	73.5 (2)	O8—P2—O7—C11	−83.4 (18)
C16—C15—S1—O1	158.4 (3)	O7'—P2—O7—C11	76 (12)
C20—C15—S1—O1	−17.9 (3)	C10—P2—O7—C11	166.5 (16)
C16—C15—S1—O2	25.5 (3)	P2—O7—C11—C12	−116 (2)
C20—C15—S1—O2	−150.8 (3)	O6—P2—O7'—C11'	55 (3)
C16—C15—S1—N1	−87.8 (3)	O8'—P2—O7'—C11'	−73 (3)
C20—C15—S1—N1	95.9 (3)	O8—P2—O7'—C11'	−72 (3)
O3—P1—O5—C2	−35 (5)	O7—P2—O7'—C11'	−94 (13)
O4—P1—O5—C2	−138 (4)	C10—P2—O7'—C11'	174 (2)
O4'—P1—O5—C2	−148 (4)	P2—O7'—C11'—C12'	168 (3)
O5'—P1—O5—C2	4(4)		

### Hydrogen-bond geometry (Å, °)

**Table:**

*D*—H···*A*	*D*—H	H···*A*	*D*···*A*	*D*—H···*A*
C5—H5A···O6^i^	0.97	2.34	3.272 (4)	162
C16—H16···O3^ii^	0.93	2.44	3.162 (5)	134
C11—H11B···O6^iii^	0.97	2.62	3.592 (19)	179.

Symmetry codes: (i) −*x*+1/2, *y*−1/2, −*z*+3/2; (ii) −*x*+1/2, *y*+1/2, −*z*+3/2; (iii) −*x*+1, −*y*+1, −*z*+2.
